# The Musashi RNA-binding proteins in female cancers: insights on molecular mechanisms and therapeutic relevance

**DOI:** 10.1186/s40364-023-00516-2

**Published:** 2023-08-25

**Authors:** Mark Sicking, Isabel Falke, Maria T. Löblein, Hans Th. Eich, Martin Götte, Burkhard Greve, Fabian M. Troschel

**Affiliations:** 1 Department of Radiation Oncology, University Hospital Münster, Albert Schweitzer-Campus 1, 48149 Münster, Germany; 2 Department of Gynecology and Obstetrics, University Hospital Münster, Albert-Schweitzer-Campus 1, 48149 Münster, Germany

**Keywords:** Musashi, RNA-binding protein, Stem cells, Breast cancer, Endometrial cancer, Cervical cancer, Ovarian cancer, Endometriosis, Radiotherapy, Inhibitors

## Abstract

RNA-binding proteins have increasingly been identified as important regulators of gene expression given their ability to bind distinct RNA sequences and regulate their fate. Mounting evidence suggests that RNA-binding proteins are involved in the onset and progression of multiple malignancies, prompting increasing interest in their potential for therapeutic intervention.

The Musashi RNA binding proteins Musashi-1 and Musashi-2 were initially identified as developmental factors of the nervous system but have more recently been found to be ubiquitously expressed in physiological tissues and may be involved in pathological cell behavior. Both proteins are increasingly investigated in cancers given dysregulation in multiple tumor entities, including in female malignancies. Recent data suggest that the Musashi proteins serve as cancer stem cell markers as they contribute to cancer cell proliferation and therapy resistance, prompting efforts to identify mechanisms to target them. However, as the picture remains incomplete, continuous efforts to elucidate their role in different signaling pathways remain ongoing.

In this review, we focus on the roles of Musashi proteins in tumors of the female – breast, endometrial, ovarian and cervical cancer – as we aim to summarize current knowledge and discuss future perspectives.

## Introduction

Therapy for female malignancies including breast, ovarian, cervical, and endometrial carcinoma has significantly improved over the last years. Multimodal approaches, including surgery, radiation therapy, chemotherapy, hormone therapy and immunotherapy have substantially reduced morbidity and mortality. However, therapy resistance, relapse and systemic spread remain major challenges [1].

In all female tumor entities, cancer stem cells (CSCs) have been identified as key drivers of cancer progression, metastasis and resistance to therapy [2]. CSCs express specific markers such as ALDH, CD133 and multidrug resistance efflux systems and possess enhanced self-renewal capacities [3]. Targeting CSC-like cells has been described as a key priority for personalized therapeutic approaches in patients with female cancers to improve outcomes [2].

The Musashi (MSI) proteins belong to the RNA binding protein family and, as such, influence a multitude of cellular processes via their RNA interaction partners. Current research increasingly focuses on their role in different cancer entities given their association with stem cell-like features. First studies indicate that targeting the MSI proteins may be a viable approach to attenuate CSC characteristics [4].

In this review we summarize the current knowledge regarding Musashi-1 and Musashi-2 in malignancies of the female. We discuss the potential therapeutic relevance of Musashi dysregulation for cancer therapy and also point to ongoing efforts to inhibit Musashi function in cancer.

## RNA binding proteins and the Musashi family – a short background

RNA binding proteins (RBPs) determine the fate of corresponding RNA binding partners by mediating transportation, stabilization, translational speed or modification of the RNA. Recognition and binding of RNA is mediated by specific RBP recognition motifs which interact with corresponding individual sequences in the RNA. Depending on their interaction partners, RBPs may impact key cellular functions, e.g., proliferation, migration, metabolism, ion transport, and signaling [5]. More than 4200 RBPs have been identified in mammalians [6–8] and RBP dysregulations have been associated with more than 160 disease entities [9]. Notably, these proteins are often dysregulated during carcinogenesis [10]. Based on their trigger function for the disease, RBP can serve as prognostic markers as well as potential therapeutic targets [11–15].

**Fig. 1:**
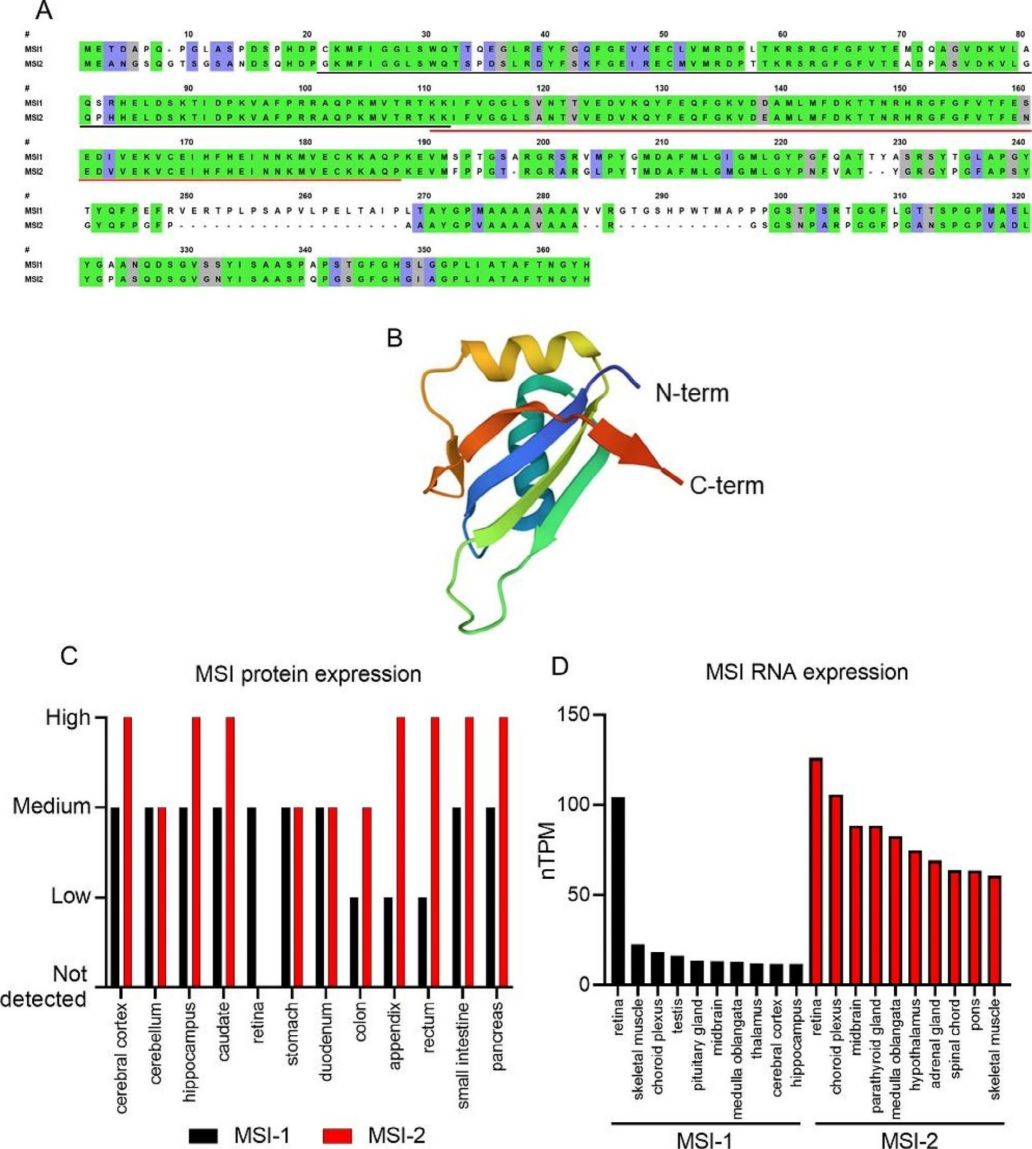
Comparison of Musashi sequences, structure and expression profile. (**A**) Alignment of MSI-1 and MSI-2 performed using www.ebi.ac.uk [38]. Green colored amino acids are identical between MSI-1 and MSI-2 and blue colored amino acids indicate strongly similar properties. Grey amino acids indicate weakly similar properties. The total sequence identity is 68,8% between the two proteins. The two RNA binding motifs are underlined in black and red, respectively. Figure was modified based on Lan et al. 2020 [19]. (**B**) Crystal structure of the human RBD 1 of MSI-2, in a resolution of 2.1 Å. Structure based on the PDB-data 6NTY by Lan et al. 2020 [19]. Both RBD (RBD1 is shown here) are formed by beta-strand parts and alpha helical domains. The color gradient follows the structure from blue (N-terminal) to red (C-terminal). (**C**) Protein expression profile for different tissues based on the dataset of the human protein atlas webpage [24–28]. Tissues in which MSI-1 (black) was detectable were selected and MSI-2 is shown in red as comparison. MSI-2 was expressed in more tissues as shown in this graph. Annotation of the expression level was based on immunohistochemically stained tissues. The specific process is detailed in the webpage. (**D**) MSI-1 and MSI-2 RNA expression profile in the ten strongest-expressing tissues. Data were taken from the database the human protein atlas [24–28]. The normalized expression (nTPM) is created by the combination of HPA and GTEx transcriptomics datasets. Detailed information about data collection and normalization are available on the webpage

The family of Musashi (MSI) proteins is small, consisting of only MSI-1 and MSI-2 with their corresponding isoforms. One shortened isoform is known for MSI-1, while there are multiple isoforms of MSI-2. A differential understanding of the isoforms is in its infancy, but first studies suggest that there are different expression patterns and functions between the isoforms [16, 17]. The main form of MSI-2 shows a 69% sequence identity to MSI-1 with an even higher overlap in the functional RNA binding domains (RBD, Fig. 1A, B) [18–20]. Hence, it is assumed that the RNA binding partners are largely similar for both proteins [21–23].

While database research for protein expression shows a robust, non-tissue-specific expression profile for MSI-2, strong MSI-1 expression was only detected in parts of the brain, retina, digestive system and pancreas (Fig. 1C) [24–28]. However, studies also demonstrated MSI-1 protein expression in testicular, spermatozoa, endometrial, ovarian and breast cancer tissue, suggesting that MSI-1 is actually present in more tissues than described in databases [29–32]. mRNA expression shows wider distribution and higher RNA expression for MSI-2 compared to MSI-1. Notably, the central nervous system is strongly overrepresented among the ten highest-expressing tissues. Both proteins were initially investigated in these tissues [18, 33, 34]. Generally, MSI levels were found to be reduced in differentiated cells compared to undifferentiated progenitor cells, pointing to the MSI function as a differentiation factor [35–37].

**Fig. 2:**
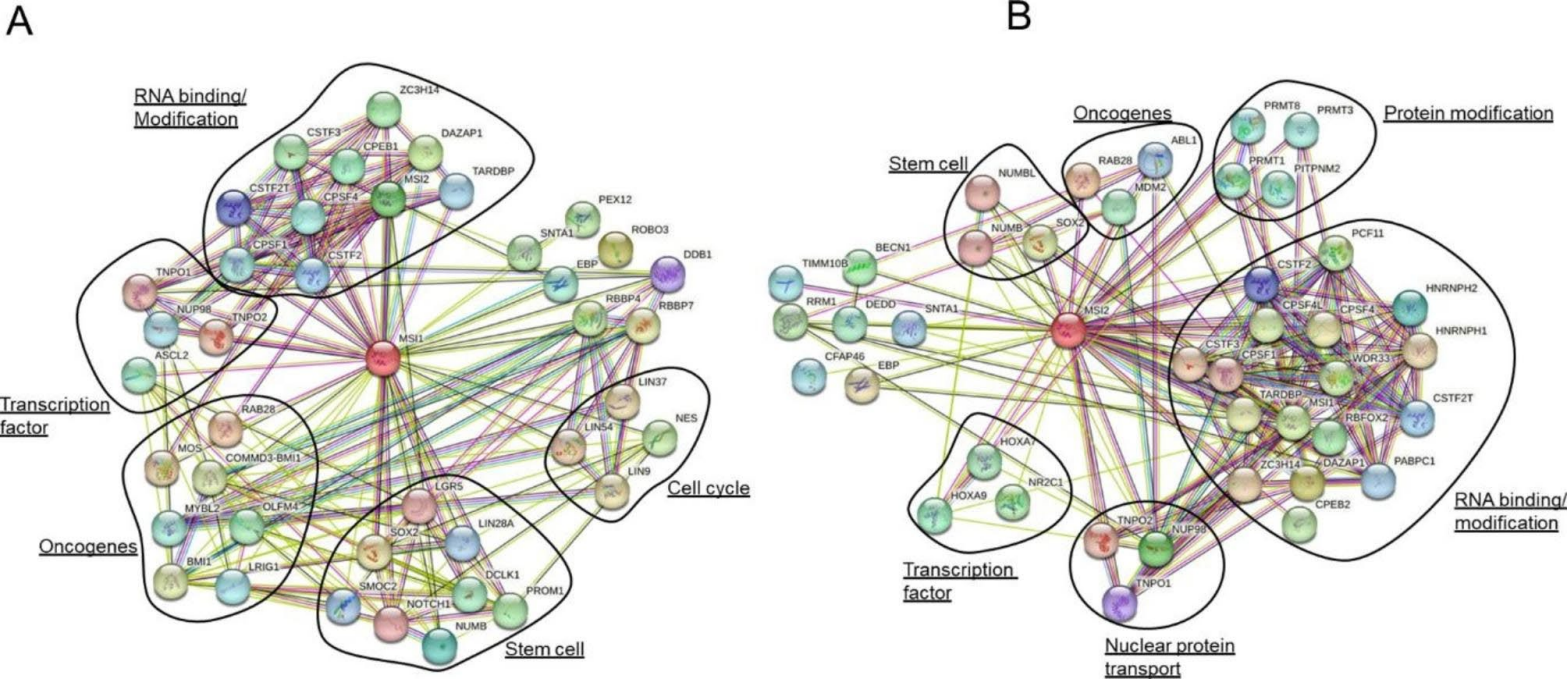
Interaction network of MSI1 and MSI2 via STRING analysis. MSI-1 (**A**) and MSI-2 (**B**) regulatory networks as identified via the STRING network analysis (version 11.5) [39–41]. The associated proteins were grouped by their function. The network is based on all available interaction sources and is limited to 40 first shell interactors. Uncircled proteins cannot be sorted into one of the specific groups

Using a STRING database analysis, we aimed to illustrate the regulatory networks of MSI-1 and MSI-2 (Fig. 2) [39]. Among 40 direct network partners independently identified for MSI-1 and MSI-2, 40% of partners were identical between MSI-1 and MSI-2 underlining close relation. For MSI-1, prominent stem cell markers on the list included, among others, NUMB, NOTCH1, PROM1 (CD133) and SOX2. For MSI-2, NUMB, and SOX2 were similarly associated.

## Musashi proteins in female malignancies

**Fig. 3:**
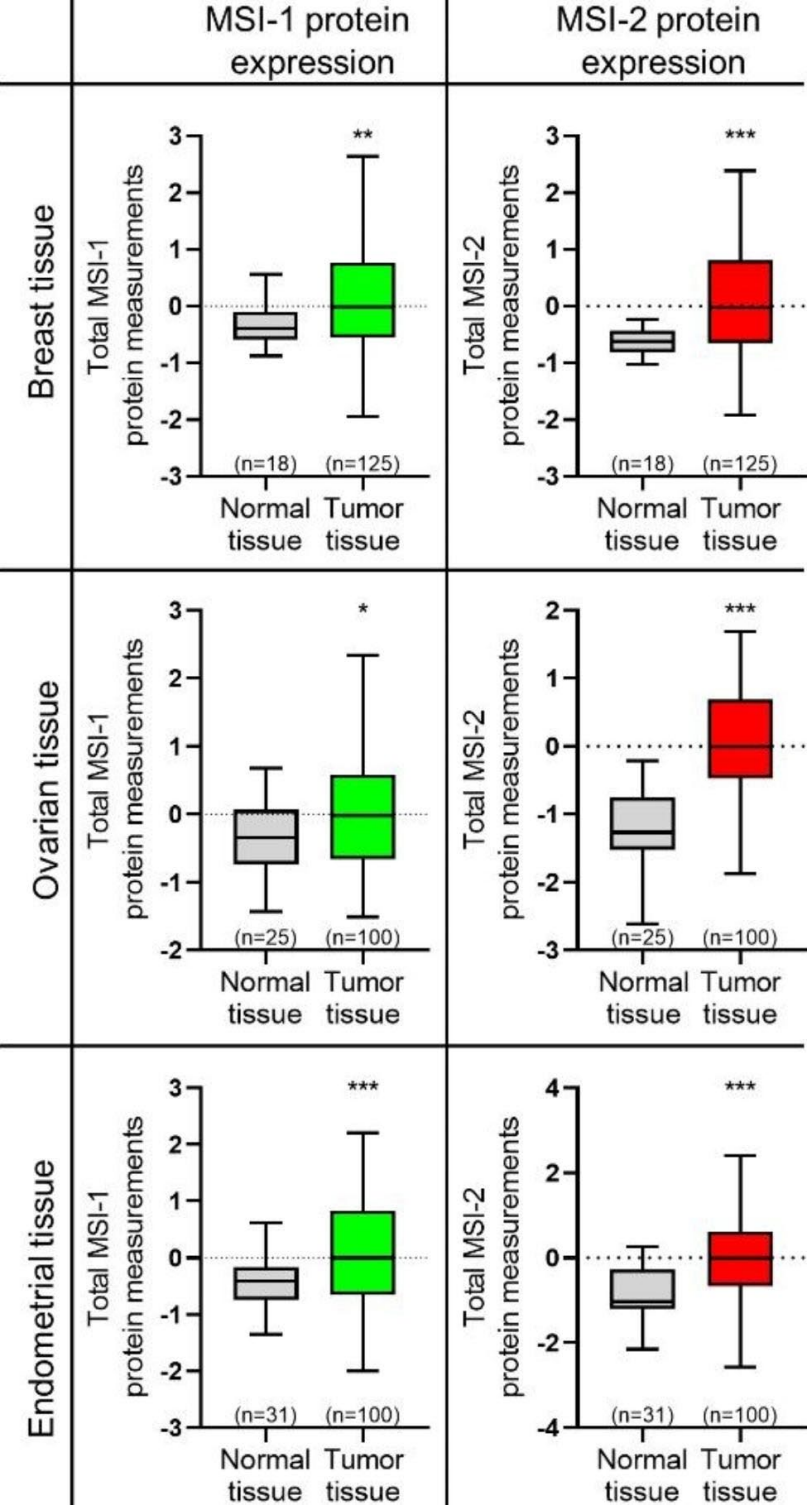
Expression of MSI-1 and MSI-2 in patient tissue samples. Protein Expression of MSI-1 and MSI-2 in different female cancers (breast, ovarian and endometrial tissue samples). MSI-1 and MSI-2 were consistently overexpressed in cancer samples compared to healthy tissues. Visualizations were based on the University of Alabama at Birmingham Cancer data analysis Portal (UALCAN) using Clinical Proteomic Tumor Analysis Consortium (CPTAC) data [42–46]. * p < 0.05, ** p < 0.01, *** p < 0.001

Using the University of Alabama at Birmingham Cancer data analysis Portal (UALCAN), we found that both MSI-1 and MSI-2 were upregulated in breast, ovarian, and endometrial cancer tissues compared to their respective healthy controls (Fig. 3) [42, 43]. Data were based on the Clinical Proteomic Tumor Analysis Consortium (CPTAC) proteomic analyses.

### Breast cancer

Breast cancer is the most common malignancy in females worldwide. While prognosis is generally favorable, cancer relapse and metastasis remain major challenges. Additionally, specific subgroups, including inflammatory or triple-negative (TNBC) cancers are associated with a worse prognosis and limited therapeutic options. Subsequently, addressing therapy resistance remains challenging in breast cancer therapy, prompting multiple studies of MSI function in this setting [47].

#### Musashi-1-specific findings

*Cancer stem cells*: MSI-1 was first established as a CSC marker in breast cancer and glioma given its co-localization with NOTCH-1 [48, 49]. These observational findings were later validated when breast cancer cells were sorted by flow cytometry, and MSI-1 was overexpressed 6-fold in side population cells [50]. Similarly, MSI-1 was also found to be strongly expressed in spheres of normal mammary tissue [51]. Further investigations continued to use MSI-1 as a CSC marker [52–55].

As with other malignancies, the MSI-Notch axis is a key mediator between MSI-1 expression and the stem cell phenotype [30, 56]. Multiple transduction mechanisms have been described among which the MSI-1-initiated binding and degradation of the Notch inhibitor NUMB [57] is the most prominent. However, in breast cancer, MSI-1 may also downregulate the 26 S proteasome, another Notch pathway repressor, to sustain Notch signaling [56]. Oskarsson et al. demonstrated that the extracellular matrix protein Tenascin C, another stem cell marker, is co-expressed with MSI-1 and supports MSI-1-dependent notch signaling [53, 58].

After Musashi knockdown, multiple additional CSC characteristics were downregulated in breast cancer, including the AKT/PI3K pathway, CD133, pERK, tumor spheroid formation, CD44, and HES2 [30, 57, 59]. It remains unclear whether these findings are due to more direct transduction mechanisms between MSI proteins and individual CSC characteristics or to a general loss of CSC phenotype following reduced Notch signaling.

*Expression*: MSI-1 is frequently expressed in more than half of 20 representative breast cancer cell lines [57]. In comparison with healthy tissues, MSI-1 was found to be upregulated in 40% of breast cancers [60]. Another study demonstrated even more widespread upregulation compared to normal tissues [61]. Using methylation analyses in primary breast cancer specimens, Kagara and colleagues found that MSI-1 promotor methylation (which was inversely correlated with gene expression) was lowest in TNBC, similar to other stem cell markers, including CD44 and CD133 [62–64]. Finally, Forouzanfar and colleagues demonstrated that MSI-1 expression depends on miR-125b in breast cancer [65].

*Proliferation*: Wang et al. overexpressed MSI-1 in mammary epithelial cells via viral transduction. They demonstrated that MSI-1 is a proliferative factor connected to increased proliferin-1 and decreased Dickkopf-3 secretion, ERK pathway activation and enhanced Notch signaling [66]. In a subsequent publication the group found xenograft tumor growth (in nude mice) substantially diminished after MSI-1 knockdown [57].

Nahas and colleagues demonstrated that MSI-1 stabilized the tachykinin-1 mRNA, which supported proliferation. Similar to others, they also noted an antiproliferative effect once MSI-1 was knocked down [67]. The group also found that antiproliferative signaling molecules including p16, p53 and p21 were upregulated upon MSI-1 knockdown [59]. Our group demonstrated that apoptosis was induced after MSI-1 and MSI-2 knockdown. Simultaneously, colony formation as a readout for proliferation was decreased. Additionally, a consistent reduction of cells in the S phase was seen in cell cycle analyses at 24, 48 and 72 h after MSI-1 and MSI-2 knockdown [30].

*Therapy resistance*: In vitro, breast cancer cells were more sensitive to irradiation after knockdown of MSI-1 or knockdown of MSI-1 and MSI-2 [30, 68]. Explanations included increased level of the radiosensitizer p21, a direct MSI target, and decreased expression of the DNA-dependent protein kinase catalytic subunit (DNA-PKcs), of the radiation resistance marker Epidermal Growth Factor Receptor (EGFR) and of CSC features in general, given their association with radioresistance. While MSI-1 low-expressing breast tumors were more likely to respond to chemotherapy in database analyses, no chemosensitizing effect was seen after knockdown in vitro [68].

*Epithelial-mesenchymal transition*: Katz et al. found that both MSI proteins were vital for blocking the epithelial-mesenchymal transition (EMT) [60]. MSI overexpression kept cells in the epithelial state by blocking translation of Jagged-1 and initiating splicing changes typical for luminal cells. Meanwhile, reduction of MSI-1 and MSI-2 increased loss of epithelial identity. MSI-1 was co-localized with E-cadherin, a key epithelial marker, in immunohistochemistry measurements. Finally, MSI-1 and MSI-2 were higher expressed in predominantly luminal breast cancer cell lines compared to basal cells, a finding reproduced by another group [65]. Katz et al. also found that MSI-1 overexpression decreased migration and MSI-2 overexpression reduced ductal branching [60]. Increased invasion and migration was also found by our group after knockdown of MSI-1 and MSI-2 in TNBC [30], but migration was reduced after MSI knockdown in inflammatory breast cancer [69].

*Metastasis*: Wang et al. found MSI-1 overexpressed in nodal metastases compared to the primary tumor [57]. Bi and colleagues demonstrated that MSI-1 expression was strong in breast cancer subpopulations with high metastatic potential and increased cell invasion and circulating tumor cells, potentially by downregulating TIMP3 [61]. The authors demonstrated that MSI-1 knockdown reduces invadopodia formation, subsequently decreasing metastatic ability. Another group demonstrated that Tenascin C was key to forming a metastatic niche in breast cancer and was co-expressed with MSI-1. When the authors subsequently performed MSI-1 knockdown, the metastatic ability of breast cancer cells to the lung was substantially decreased [53]. Using open-source data, our group found that low MSI-1-expressing tumors demonstrated better distant metastasis-free survival than high MSI-1-expressing tumors in breast cancer [68].

*Prognostic relevance*: Wang and colleagues found that strong MSI-1 expression in primary breast cancer tissues was associated with reduced survival [57]. Using available sequencing data, our group showed that high MSI-1 levels were associated with reduced disease-free survival and tended to be related to shorter overall survival (OS) [68].

**Fig. 4:**
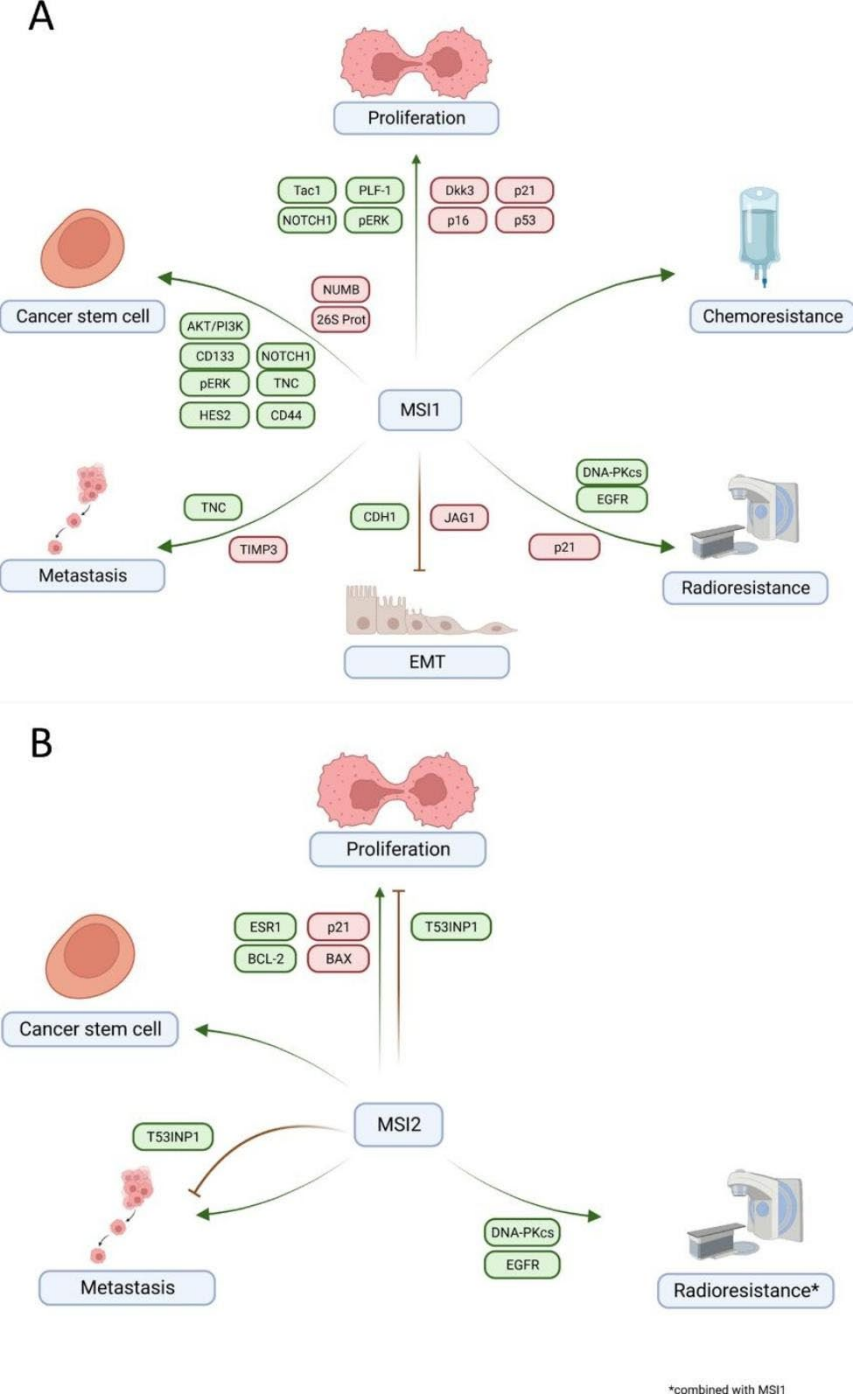
Effect of MSI-1 and MSI-2 on cell behaviour in breast cancer. Schematic overview of MSI-1 (**A**) and MSI-2 (**B**) regulatory interactions identified in breast cancer. Both proteins affect cell behavior, including metastasis, epithelial-mesenchymal transition (EMT), DNA repair, cancer stem cells and proliferation via different pathways, as indicated in the graph. For MSI-2, divergent findings have been published regarding some characteristics, as demonstrated in the graph. See text for details and literature sources. The figure was generated using Biorender.com

*Summary*: Most of the findings in breast cancer consistently point towards a tumorigenic role for MSI-1: It has been identified as a CSC marker that is overexpressed in tumor tissue. MSI-1 seems to contribute to proliferation, therapy resistance, and metastasis formation (Fig. 4A). Subsequently, survival is shorter for patients with MSI-1-overexpressing tumors. The MSI-1-EMT relationship seems to be complex. On one hand, EMT-related properties are reduced in low MSI-1-expressing cells: EMT is known to be associated with CSCs as well as wnt and Notch signaling, both of which were suppressed after MSI-1 knockdown [70]. EMT has also been associated with therapy resistance and metastasis formation, which were also reduced after MSI-1 knockdown [71]. On the other hand, Katz et al. report that reduced MSI-1 expression may induce EMT, a seemingly contradictory finding [60]. Based on studies in lung cancer, the explanation seems to be that MSI protein knockdown stimulates a “partial EMT” [72]. Specifically, some pro-EMT signaling is increased after MSI knockdown (e.g., upregulation of Jag1, Twist, ZEB1, ZEB2, and FOXC2 and downregulation of E-cadherin) [60, 72]. Thus, MSI knockdown leads to some EMT-like cellular changes, including decreased cell-cell interactions and a change in cell phenotype. However, MSI knockdown simultaneously induces key anti-EMT signaling, including downregulation of Vimentin, SNAIL and SLUG as well as the notch and wnt pathways [72], thus blocking a full EMT. Hence, the functional pro-tumorigenic properties (e.g., formation of metastases) normally associated with EMT are not seen after MSI-1 knockdown. In fact, low MSI-1-expressing tumors form metastases at a substantially lower rate compared to high MSI-1-expressing cancers. Thus, in sum, anti-tumorigenic signaling with decreased proliferation, metastasis, therapy resistance and CSC phenotype seems dominant despite partial EMT after MSI-1 targeting.

#### Musashi-2-specific findings

MSI-2 has been investigated to a much smaller degree compared to MSI-1 and remains more controversial:

*Cancer stem cells*: Similar to MSI-1, MSI-2 levels have been found to be overexpressed in mammospheres and MSI-2-overexpressing cells show increased capability to form mammospheres [73]. As mammosphere formation is associated with CSC features, this indicates that MSI-2 may also be involved in CSC maintenance [74]. MSI-1 and MSI-2 targeting resulted in reduced CSC characteristics in TNBC [30].

*Expression*: MSI-2 has been found to be upregulated in 50% of breast cancers [60]. Within breast cancers, it is increased in estrogen receptor positive breast cancer and decreased in TNBC to a degree that it might be expressed at a lower level than in their adjacent normal tissue [17, 75]. One study found a direct correlation between expression of the estrogen receptor 1 (ESR1) and MSI-2 using genomic analyses and immunohistochemistry, hypothesizing that MSI-2 is a direct upstream regulator of ESR1 [68].

*Proliferation*: ESR1 is a known proliferative marker [76]. Kang et al. found ESR1 level decreased after MSI-2 knockdown, resulting in repressed clonogenic potential [75]. Choi et al. demonstrated that MSI-2 knockdown induced cell cycle arrest, while increasing apoptosis in breast cancer cells [77]. In parallel, p21 and BAX increased, and BCL-2 decreased. Subsequently, MSI-2 knockdown substantially decreased colony formation, while MSI-2 overexpression increased clonogenic capacity. Holzapfel et al. similarly reported increased proliferation after MSI-2 overexpression in hormone sensitive and TNBC cells and suggested consideration of MSI-2 as a therapeutic target [73]. Our group found reduced proliferation after dual knockdown of MSI-1 and MSI-2 in TNBC [30].

Conversely, Li et al. showed increased viability and proliferation after MSI-2a knockdown in triple-negative breast cancer cells (the other major isoform, MSI-2b, elicited no changes at all). Overexpression of MSI-2a in xenograft tumors was reported to abrogate tumor growth [17].

*Therapy resistance, metastasis, and Prognostic Relevance*: While dual knockdown of MSI-1 and MSI-2 reduced radioresistance [30], no MSI-2-specific studies have investigated therapy resistance. Li et al. found that low expression of isoform MSI-2a is associated with increased rates of metastasis, while its high expression decreased breast cancer metastasis formation [17]. The authors hypothesized that decreased expression of tumor suppressor TP53INP1, stabilized via MSI-2, was responsible for these effects. Conversely, Holzapfel et al. reported increased invasion and migration after MSI-2 overexpression [73]. Using the Kaplan Meier plotter tool, Kang et al. demonstrated that low MSI-2 expression was associated with substantially worse outcomes (p < 0.001) [75]. Similarly, Li et al. also found MSI-2 to be a positive prognostic marker [17].

*Summary*: The scarcity of data and their inconsistency complicate a clear interpretation of the role of MSI-2 in breast cancer (Fig. 4B). The majority of reports indicate that MSI-2 repression leads to decreased proliferation and therapeutic resistance and may downregulate stem cell features. Surprisingly, MSI-2 seems to be a positive prognostic marker on first glance. This finding is linked to MSI-2 overexpression in the (good-prognosis) estrogen receptor positive tumor group compared to, e.g., TNBC tumors. In fact, once the survival analysis is run in ER-positive and ER-negative tumors separately using the same tool as in previous publications (the Kaplan Meier plotter), any prognostic relevance entirely vanishes (HR = 0.99 and *p* = 0.88; HR = 1.02 and *p* = 0.9, respectively) [78]. This indicates that any positive prognostic value attributed to MSI-2 hinges exclusively on its expression disparity between ER-positive and -negative tumors. Thus, even Kang et al., who found the positive association, still primarily recommend evaluation of MSI-2 as a therapeutic target, not as a marker of good prognosis [75].

However, questions remain regarding the findings provided by Li et al. Their study seems to contradict the other MSI-2 breast cancer studies and numerous studies in other tumor entities that found a tumorigenic role for MSI-2 [72, 79, 80]. It remains to be seen whether the individual manipulation of the MSI-2a isoform as opposed to a general MSI-2 knockdown is sufficient explanation for this substantial difference in results.

### Ovarian cancer

Ovarian cancer is associated with a poor prognosis among gynecologic cancers. Therapeutic options are often limited and therapy resistance is a major challenge considering late diagnosis of malignancy and limited therapeutic efficiency [81].

There are only few, but promising data concerning ovarian cancer and the Musashi proteins. Two studies demonstrated a high expression of MSI-1 in ovarian cancer tissues compared to healthy controls. Within tumor tissues, MSI-1 expression is correlated with higher tumor stages, CA-125 level, tumor mass and chemotherapy resistance [82, 83]. For MSI-2, a higher expression in tumor compared to healthy tissues and a correlation between high level of MSI-2 and advanced tumor grades were also confirmed [84]. Inhibition of both MSI-1 and MSI-2 led to improved chemotherapy response to paclitaxel [82, 84]. Our group further demonstrated that a simultaneous knockdown of both MSI proteins in ovarian cancer cells leads to better radiotherapy response in vitro [31]. Therefore, high MSI-1 and MSI-2 expression may complicate therapeutic success in ovarian cancers. However, as a limitation, all studies either consisted of small study groups (110 and 257 patients, respectively) or investigations were based on cell culture experiments. Therefore, further research is needed to validate these findings in vivo or in more complex in vivo-inspired 3D cultures or organoids.

Additionally, database analyses in ovarian cancer patient samples revealed that both MSI-1 and MSI-2 play important roles in many signaling pathways. Not only do MSI-1 and MSI-2 regulate each other, but are also of regulatory importance for the NOTCH signaling pathway, P21, MYC and ALDH subtypes [31]. These targets are well known as parts of oncogenic signaling pathways and CSC-associated genes [85]. The impact MSI proteins have on all these pathways in vivo has yet to be investigated.

In sum, although there is a lack of data, MSI targeting merits interest in ovarian cancer given anti-tumorigenic results.

### Endometrial cancer

In industrialized countries, endometrial cancer (EC) is the most common malignancy of the female reproductive tract [86]. While early stage-EC are associated with a good prognosis, 5-year-survival is strongly reduced in patients with distant metastases or cancer recurrence [87].

In 2008, our group found increased MSI-1 expression in EC tissues. EC MSI-1 expression presented as nuclear and cytoplasmic staining. Immunofluorescence microscopy further revealed co-localization of MSI-1 with CSC markers Notch-1 and telomerase [88]. Two studies investigating endometrioid adenocarcinoma (EAC), the most common histological subtype (85%), confirmed increased MSI-1 expression [89, 90]. High MSI-1 expression was associated with poor survival and advanced tumor stage, grade and vascular invasion [13].

MSI-1 was overexpressed in CSCs in endometrial cancer tissue [91]. Experimental knockdown of MSI-1 in Ishikawa EC cells led to an increase in p21 and decrease in Cyclin B1, Notch-1 and Hes-1 levels. Subsequently, antiproliferative effects on cell cycle progression and apoptosis were seen [91].

In a follow up study [92], MSI-1 silencing restricted cancer cell proliferation and sensitized cells to irradiation in Ishikawa (EC type 1) and KLE (type 2) cells. CSC markers TERT (telomerase reverse transcriptase) and the Numb/Notch pathway were also downregulated. DNA repair protein DNA-PKcs was downregulated, while the antiproliferative marker p21 was increased. Associations were also found in database analyses of primary tissue samples [92].

In vivo, MSI-1-depleted tumors demonstrated significantly reduced growth compared to control tumors. Immunohistochemical staining revealed more apoptotic and less mitotic areas in these tumors. TERT expression was decreased [92].

Additionally, MSI-1 knockdown resulted in decreased radioresistance in both cell lines with a stronger effect in radiosensitive KLE cells [93]. Contrary to other female malignancies, no chemosensitization was seen. Overall, silencing of MSI-1 presented as a potential therapeutic option regarding tumor growth and radiation therapy in MSI-1 expressing endometrial cancers [92]. No further research regarding the impact of MSI-2 on endometrial cancer is currently available.

### Cervical cancer

Cervical cancer (CC) is the fourth most frequently diagnosed cancer and the leading cause of female cancer death in 36 countries. High risk human papillomavirus (HPV) is the main risk factor [86]. Most cases of CC occur in less developed countries reflecting limited availability of screening programs and HPV vaccines. Despite reduced incidence and mortality rates in developed countries, cancer recurrence, metastasis and therapy resistance remain challenging [86, 94]. CSCs contribute to the tumorigenic potential in development, metastasis, and recurrence of cancer [95].

MSI-1 was found to be highly expressed in CC tissues in two studies [96, 97]. High expression was correlated with poor OS, poor progression-free-survival (PFS) and cancer recurrence [97].

Experimental overexpression of MSI-1 enhanced tumor formation and cell proliferation in vitro and in vivo. MSI-1 was shown to promote CC proliferation by accelerating G0/G1-S cell cycle transformation via targeting of cell cycle proteins p21, p27 and p53 [96]. The group also suggested that MSI-1 inhibits cancer cell apoptosis and therefore promotes tumor formation in vitro and in vivo. In MSI-1-overexpressing cells upregulation of PI3K and p-AKT and downregulation of PTEN led to reduction of the pro-apoptotic protein BAK. A rescue experiment was performed to confirm the critical role of BAK in the MSI-1-PTEN-AKT pathway [98].

MSI-2 expression is increased and significantly associated with FIGO stage and lymph node metastasis in CC tissues. OS and PFS were significantly decreased in patients with higher MSI-2 expression compared to patients with lower MSI-2 expression. The prognostic value of MSI-2 expression was further analyzed in subgroups: High MSI-2 expression, OS and PFS were significantly increased in patients with FIGO stage ≤ 1 (early stages) and grade 3 tumors, indicating that MSI-2 may contribute to early-stage CC progression. Accordingly, knockdown of MSI-2 inhibited invasion and migration of CC cells in two different CC cell lines [79].

Mechanistically, it was shown that MSI-2 promotes CC growth, invasiveness, and sphere formation by downregulation of the protooncogene c-FOS. MSI-2 was demonstrated to be negatively regulated by p53: Natural antibiotic Mithramycin A increased p53 and upregulated miR-143 and miR-107. These tumor suppressor miRNAs directly bound and decreased MSI-2 expression, resulting in downregulation of c-FOS and suppressed invasion as well as proliferation of CC cells. Increase of miR-143/miR-107 by Mithramycin A via activation of p53 may be a novel therapeutic approach for CC [99].

In 2020, another group showed that knockdown of long non-coding RNA miR-4435-2HG suppressed proliferation, migration, and invasion of CC cells via regulating the miR-128-3p/MSI-2 axis in vitro. miR-4435-2HG expression was upregulated in CC tissue as well as in cell lines and was shown to target miR128-3p which itself suppressed MSI-2. Both downregulation of miR-128-3p and upregulation of MSI-2 reversed the inhibitory effects of miR-4435-2HG knockdown on proliferation, migration, and invasion [100].

### Endometriosis

Endometriosis is an estrogen-dependent disease characterized by the growth of endometrial tissue outside the cavum uteri [101]. Affected patients suffer from a broad range of pain symptoms and reduced fertility. It has been estimated that up to 10% of women of reproductive age may be affected by the disease [102]. Endometriosis shares pathophysiological similarities to cancer progression despite its benign pathology, involving dysregulation of the immune system, invasive growth, and aberrant angiogenesis [103]. Moreover, a positive association of endometriosis with ovarian and thyroid, and a negative association with cervical cancer has been reported [104]. We and others have postulated that an aberrant stem cell function may be linked to the pathogenesis of endometriosis, as classical etiological concepts such as retrograde menstruation or coelomic metaplasia are compatible with an increased persistence of stem cells and their higher developmental plasticity at ectopic sites [103, 105].

Indeed, studies from our group have suggested a role of the notch signaling pathway in endometriosis, since notch-1 expression in endometrial glands is significantly higher in the eutopic endometrium of patients with deep infiltrating endometriosis compared with controls [106]. Furthermore, in vitro data in the endometriotic cell line 12Z and in primary endometriotic stroma cells demonstrated that attenuation of the Notch signaling pathway with gamma-secretase inhibitors reduced viability and the stem cell phenotype of endometriotic cells, whereas apoptosis was enhanced [107].

An early study found that MSI-1 was co-localized with Notch-1 and telomerase in endometrial tissue [88]. Immunohistochemistry of patient tissues revealed that MSI-1-expressing stromal cell numbers were significantly increased in endometriotic tissue compared to healthy secretory endometrium. A study on ovarian endometriosis identified MSI-1 expression in stromal colony forming units [108]. These data prompted us to perform an siRNA double-knockdown in the endometriotic cell line 12Z and primary endometriotic stroma cells in vitro to study a potential mechanistic involvement of MSI-1/2 in endometriosis [109]. MSI-1/2-double-knockdown increased apoptosis and necrosis and reduced cell proliferation, ALDH activity, the side population and spheroid formation as a stem cell activity readout. At the molecular level, a downregulation of stemness-associated protein expression (including Hes-1 and Notch-2) and an upregulation of p21 expression were observed after MSI depletion, suggesting that MSI-1/2 may promote endometriosis by enhancing cell proliferation, viability and the stem cell phenotype [109]. It is noteworthy that some natural therapies may exert their anti-endometriotic effect at least partially by affecting MSI function, as treatment of the endometrial stroma cell line St-T1b with resveratrol significantly reduced MSI-1 expression, while it decreased cell viability and cell migration and increased apoptosis in vitro [110]. As natural therapies have recently gained considerable attention as potential alternatives to side-effect-prone endocrine approaches, this therapeutic route may deserve further investigation [111].

Overall, high MSI expression seems to be tumorigenic and prognostically unfavorable in female malignancies, based on its influence on cellular mechanisms promoting malignancies as summarized in Table 1. Targeting MSI proteins seems to decelerate cell growth and make cells more susceptible to cancer therapy. However, not all effects are uniform between the different entities or even within a single malignancy (e.g., ovarian cancer proliferation).

**Table 1:** Summary of the effects mediated by MSI-1 or MSI-2 in the different female cancer entities

Tumor entity	Characteristics	Musashi-1	Musashi-2
Breast cancer	Expression	Overexpressed in cancer [61]	Overexpressed in cancer [60] except for TNBC [17]
	Cancer stem cell	CSC marker [50]	CSC marker [73]
	Proliferation	Induces proliferation [66]	Induces or blocks proliferation, unclear [17, 66]
	Therapy	Marker of radioresistance [68]	Marker of radioresistance at least in combination with MSI-1 [30]
	Prognosis	Negative prognostic marker [57]	Positive prognostic marker [75] No significance once analyzed separately for estrogen receptor positive and negative tumors
	Future perspective	In vivo studies needed	Clarification regarding role as oncogene or tumor suppressor necessary
Ovarian cancer	Expression	Overexpression in cancer [83]	Overexpression in cancer [84]
	Cancer stem cell	CSC marker [31]	CSC marker [31]
	Proliferation	Anti-proliferative in one of two cell lines [31]	Anti-proliferative in one of two cell lines [31]
	Therapy	Marker of radio- and chemoresistance [82]	Marker of radio- and chemoresistance [84]
	Prognosis	Negative prognostic marker [83]	N.a.
	Future perspective	In vivo studies needed	In vivo studies needed, prognostic relevance unclear
Endometrial cancer	Expression	Overexpressed in cancer [89, 90]	N.a.
	Cancer stem cell	CSC marker [92]	N.a.
	Proliferation	Induces proliferation [92]	N.a.
	Therapy	Marker of radioresistance [92]	N.a.
	Prognosis	Negative prognostic marker [13]	N.a.
	Future perspectives	Translational assessment necessary	No prior investigation
Cervical cancer	Expression	Overexpressed in cancer [96, 97]	Overexpressed and associated with lymph node metastasis [79]
	Cancer stem cell	N.a.	N.a.
	Proliferation	Enhances proliferation in vivo an in vitro [79]	Enhanced invasion, proliferation and migration in two cell lines [79]
	Therapy	N.a.	N.a.
	Prognosis	Negative prognostic marker [97]	Negative prognostic marker [79]
	Future perspectives	Therapeutic relevance unclear, in vivo studies needed	Therapeutic relevance unclear, in vivo studies needed

N.a., no data available

## Future directions

We believe the following three aspects would be most relevant for future research:

### Musashi-2 in breast and endometrial cancer: friend or foe?

Due to a substantial uncertainty regarding the role of Musashi-2 in both breast and endometrial cancer, further study is clearly needed.

In breast cancer, the existing findings support contradicting statements on MSI-2-related effects regarding proliferation and cell viability, as discussed above. Most studies suggest MSI-2 confers pro-metastatic and pro-proliferative properties, while Li et al. found that MSI-2a – the main MSI2 isoform in breast cancer – may decrease metastases and proliferation. Isoform-specific knockdowns may help answer whether different MSI-2 isoforms have diverging effects on breast cancer cells, as suggested by Li et al. Additionally, future work in primary cell cultures, tumor organoids or in vivo work may help solve this contradiction. Finally, MSI-2 (or isoform-specific) knockout models may also offer more authoritative data.

Surprisingly, we were unable to find any study on MSI-2 in endometrial cancer. Database analyses suggest a robust expression of MSI-2 in this cancer entity, offering intriguing potential for future exploratory analyses.

### Mechanistic analyses

While many of the discussed studies have described important MSI-mediated morphologic changes, underlying mechanisms have oftentimes not conclusively been described. While it is tempting to rely on mechanistic investigations in other tumor entities, this should only be done with caution given that, for example, MSI-2 is a *positive* prognostic marker in clear cell renal cell carcinoma, but a *negative* prognostic marker in cervical cancer [79, 112]. This suggests that tumor-specific factors determine the relevance MSI-dependent mechanisms. Thus, we believe that mechanistic study of MSI-related effects in female cancers should be prioritized in future research. Here, the availability of large-scale sequencing analyses to analyze both direct binding partners and general gene expression modifications is likely to facilitate an improved understanding. These findings may also help determine otherwise contradictory functional assay results.

### Targeting MSI proteins – use of inhibitors

Largely promising results following MSI downregulation have sparked an increased push to identify MSI inhibitors. Different high throughput studies were performed within the last five years, and multiple competitive inhibitors of the Musashi RNA binding domains were found. Inhibitors may specifically target MSI-1, MSI-2, both, and / or additional targets:

- (-)gossypol and gossypolone, cottonseed derivatives, bind to the RBD1 of MSI-1 [113]. Studies in breast and colon cancer cell lines showed anticancer effects and colon cancer growth was reduced in a mouse xenograft model upon (-)gossypol treatment. Notably, (-)gossypol also inhibits the proteins of the Bcl-family as well as the RNA binding protein mouse double minute 2 (MDM2), likely contributing to anti-tumorigenic effects [113–121]. Gossypolone, the derivate of (-)gossypol, was more recently identified as another, more potent MSI inhibitor from the same family [118, 122].
- Luteolin again binds to the RBD1, subsequently inhibiting tumorigenicity including proliferation, cell viability, and colony formation. It also sensitizes glioblastoma cells to high doses of radiation and chemotherapy [123, 124].
- Ro-08-2750 also inhibits MSI-1 and MSI-2 [125]. It was additionally demonstrated to be an interesting candidate for further investigation due to its well-tolerated application in mice [126]. Myeloid leukemia cells treated with this drug show similar effects compared to an sh-RNA treatment for MSI-2 [127]. An increase in differentiation, apoptosis and an inhibition of known MSI targets was observed upon treatment with Ro-08-2750. Ro additionally inhibits the nerve growth factor (NGF), another tumor driver [128].
- Aza-9 binds MSI-1 and MSI-2, downregulating the Notch/Wnt pathway and cell while inducing apoptosis and autophagy [129].

Except for (-)gossypol, these inhibitors have not been evaluated in cancers of the female despite promising potential in other tumor entities. Should MSI1 and MSI2 be further confirmed as therapeutic targets upon additional mechanistic assessment (see above), we suggest in vitro and in vivo studies to assess their efficacy. An overview of known MSI inhibitors is displayed in Table 2.

**Table 2:** Summary of Musashi inhibitors and their effects and functions

Inhibitor	target	K_D_	tested in	Effect after treatment	additional targets	comment	source
Aza-9	MSI-1	1.2 µM	Colon cancer cell lines	Inhibited proliferation, induced apoptosis / autophagy G1 accumulation	HuR		[129, 130]
	MSI-2	0.5 µM					
(-) gossypol	MSI-1	476 ± 273 nm	Colon cancer cell lines Breast cancer cell lines Pancreatic cancer cells	Reduced Notch/Wnt signaling Increased apoptosis/autophagy, reduced tumor growth in xenografts Reduced proliferation, damaged cell membrane integrity Triggered apoptosis and suppression of spheroid formation	BCL-2 family MDM2	Different clinical trials were performed	[113, 118, 121, 131, 132]
Gossypolone	MSI-1	13 ± 5 nm	Colon cancer cell lines Breast cancer cell line	Inhibited proliferation Induced apoptosis/autophagy, Inhibited tumor growth of human colon cancer cell xenografts in nude mice Suppression of DNA synthesis	BCL-2 family		[118, 122]
	MSI-2	7 ± 0.3 nm					
Ro 08-2750	MSI-1	N.a.	Bone marrow cells Murine leukemia model Chronic lymphatic leukemia	Reduced cell proliferation, increased apoptosis Reduced colony formation Reduction of viable cells	Inhibits NGF with lower K_D_ (1.7 µM)	SRF2 and SYNCRIP proteins are bound with 15–20 x higher K_D_	[125, 127, 128]
	MSI-2	12.3 ± 0.5 µM					
Largazole	MSI-2		Lung cancer and CML cell lines	Inhibited proliferation and induced apoptosis, suppressed colony formation		The amount of MSI2 mRNA is reduced after treatment	[133]
Luteolin	MSI-1	3.2 ± 0.02 µM	Glioblastoma cell lines	Inhibited proliferation, migration, colony formation and invasion	Reduces oxidative stress		[123, 124]
Palmatine	MSI-1	17 µM Kd (in vitro) 26.4 µM Kd (in cellular protein based assays)	Colon cancer cell line and xenograft model	Reduced cell growth Reduced tumor growth in mice Multiple anti-cancer functions	rpS6/NFkB/FLIP axis AURKA	First three days of treatment resulted in weight loss in mice	[134–136]
	MSI-2	67.5 µM Kd (in cellular protein based assays)					
oelic acids	MSI-1	1.2 ± 0.4 µM	HeLa Mouse model	Increased amount of miR-7 (tumor suppressor) in vitro Increased fibrosis and apoptosis in kidneys after injection		Erucic acid binds with even higher affinity to MSI1 “Musashi1 appears to act as a `nutrient sensor` turning up its activity when oleic levels are low and vice versa”	[137–139]
	MSI-2	4.7 ± 0.5 µM					

## Conclusion and perspectives

Numerous studies have provided substantial evidence to connect both Musashi proteins to tumorigenicity, therapy resistance, cancer stem cell phenotype, increased proliferation and reduced prognosis in most cancers of the female. Here, only some uncertainties remain, especially regarding MSI-2 in breast and endometrial cancer.

However, while MSI proteins are generally described as key regulators of post-transcriptional gene expression, much remains unknown regarding the specific regulatory pathways involved. In this setting, additional mechanistic studies will help close the knowledge gap regarding the interplay between MSI expression and associated functional or morphologic tumor characteristics.

MSI targeting has been identified as a promising therapeutic approach in female and other malignancies given reduction of proliferation, invasion and cancer stem cell phenotype and increase in apoptosis, autophagy and therapeutic cell eradication. Here, most findings rely on cancer cell line studies. Only few – yet promising – in vivo experiments have been performed. Additional studies will be needed to substantiate these findings.

Inhibiting the Musashi proteins has increasingly become feasible given the recent discovery of multiple inhibitors. Preliminary results demonstrate relevant anti-tumorigenic properties of Musashi inhibitors. However, most inhibitors have not been assessed in female cancers, obscuring their potential in this setting.

In sum, both the understanding of Musashi protein function and its application via targeting and inhibition remain work in progress. While prior investigations indicate promising potential, mechanistic understanding and successful translation remain complex. Ongoing and future studies will refine our knowledge of these regulators and may provide better diagnostic and therapeutic options to treat female malignancies.

## Data Availability

The datasets analysed during the current study are available as indicated in the manuscript.

## Abbreviations

CC: Cervical Cancer
CSC: Cancer stem cell
DNA-PKcs: DNA-dependent protein kinase catalytic subunit
EC: Endometrial cancer
EAC: Endometrioid adenocarcinoma
ESR1: Estrogen receptor 1
HPV: High risk human papillomavirus
MSI-1: Musashi-1
MSI-2: Musashi-2
OS: Overall survival
PABP: poly (a) binding proteins
PFS: progression-free survival
RBD: RNA binding domain
RRM: RNA recognition motif
RBP: RNA binding protein
TERT: Telomerase reverse transcriptase
TNBC: Triple negative breast cancer
